# Mast cell activation syndrome—anesthetic challenges in two different clinical scenarios

**DOI:** 10.7555/JBR.36.20220071

**Published:** 2022-05-28

**Authors:** Brianna Lide, Shane McGuire, Hong Liu, Cristina Chandler

**Affiliations:** Department of Anesthesiology and Pain Medicine, University of California Davis Health, Sacramento, CA 95817, USA

**Keywords:** mast cell disease, mast cell activation syndrome

## Abstract

Mast cell activation syndrome (MCAS) includes a group of disorders that result in the inappropriate release of inflammatory mediators from mast cells. These mediators can affect multiple organ systems and lead to significant morbidity, and possible fatality. Although reactions, typically in response to various nonspecific stimuli, are usually mild, they may put those with MCAS at increased risk of anaphylaxis. In this case report, we present two clinical scenarios of MCAS, and identify possible factors triggering mast cell mediator release. We also define a preoperative preventive pathway, outline anesthetic considerations, and discuss the management of immediate hypersensitivity reactions in patients with MCAS. Meticulous preoperative preparation, avoidance of triggers, and development of a plan to treat possible adverse organ responses are paramount of good outcomes.

## Introduction

Mast cell activation syndrome (MCAS) includes a group of disorders in which mast cell activation is the primary pathologic mechanism of disease^[1]^. MCAS subtypes include primary MCAS, secondary MCAS, and idiopathic MCAS as defined by an international allergy & immunology work group in 2012^[2]^. Primary MCAS, sometimes referred to as "clonal MCAS", results from monoclonal mast cell proliferation most commonly due to an activating mutation in protein tyrosine kinase receptor KIT^[2]^. Mastocytosis (cutaneous and systemic) is considered a primary MCAS. Secondary MCAS describes normal mast cells which are activated by an identifiable external trigger. The third subtype, idiopathic MCAS, describes an entity in which no identifiable cause of mast cell activation has been found^[1–2]^. MCAS leads to inappropriate mast cell activation resulting in release of numerous multisystem inflammatory mediators which may or may not fit the presentation of a typical allergic reaction^[1]^. Mast cell activation may be immune-regulated (*i.e.*, IgE) or non-immune-regulated *via* cytokines, G-protein coupled receptors, and physical stimuli. The result is release of preformed mediators including histamine, prostaglandins, leukotrienes, and proteases including tryptase^[1]^. Because MCAS has only recently been defined, most of the current literature available focuses on mastocytosis. Patients gave permissions for this case report, and the ethical standards of the institutional committee on human experimentation and the Helsinki Declaration were followed.

## Case reports

### Case 1

A 34-year-old woman G1P0 at 41 weeks, with inadequate prenatal care, was urgently transferred to our facility from a local midwife birthing center. Emergent cesarean was indicated due to fetal breech position. The patient had a medical history of mast cell activation syndrome with multiple anaphylaxis events in the past. The anesthesiologist was unable to obtain further detailed history and the allergy list provided was vague, including "opioid medications" and an extensive list of antibiotics. She refused regional anesthesia and the decision was made for general anesthesia (GA) with endotracheal intubation. Prior to induction, the patient was given intravenous diphenhydramine 50 mg, famotidine 20 mg, and methylprednisolone 125 mg. Rapid sequence induction was performed with propofol 200 mg and succinylcholine 120 mg. Clindamycin 900 mg and gentamicin 120 mg were given for infection prophylaxis due to a documented penicillin allergy. After delivery of a healthy infant, oxytocin 3 units were infused at 41.7 milliunits/minute. The patient required additional bolus of oxytocin 3 units for poor uterine tone and responded appropriately. A radial arterial line was placed for hemodynamic monitoring and frequent blood sampling. Pain was controlled with intravenous fentanyl boluses 50 to 100 μg. Mild hypotension was treated with phenylephrine and ephedrine but otherwise she remained hemodynamically stable. She was extubated at the end of surgery and the total time under GA was 3 hours and 15 minutes. Postoperative pain was controlled with a fentanyl PCA and PO acetaminophen. The next day, her home antihistamine, ketotifen fumarate, was restarted. Abdominal computer tomography (CT) was performed 24 hours postoperatively to evaluate ureter injury with diphenhydramine premedication. She experienced no signs or symptoms of mast cell degranulation during her intra- or postoperative course.

### Case 2

A 64-year-old woman with a complicated medical history of severe persistent asthma and mild bronchiectasis, type 2 diabetes mellitus, hypertension, and presumed idiopathic MCAS presented for elective bilateral mastectomy for left breast carcinoma. She underwent several surgeries in the past and had experienced multiple episodes of anaphylaxis. Her extensive list of allergies included local anesthetics, numerous antibiotics, and opioids. A perioperative plan was developed by the patient's allergist, pulmonologist, surgeon, and anesthesiologist. The patient was started on a preoperative treatment with cromolyn 200 mg QID for 7 days, ketotifen 0.5 mg for 7 days followed by 1 mg for 3 days. On the morning of surgery, she took levocetirizine 5 mg, montelukast 20 mg, famotidine 40 mg, and cetirizine 20 mg. No preoperative steroids were given. After GA was induced with etomidate 0.3 mg/kg and rocuronium 1 mg/kg, the patient was intubated. Intraoperative pain was controlled with methadone given in 2 mg boluses, dexmedetomidine with 1.2 μg/kg loading dose followed by infusion of 0.6 μg/(kg·hour) and ketamine infusion 4 μg/(kg·minute). The intraoperative hypertension was treated with clevidipine infusion. She was extubated at the end of the operation and there were no intraoperative complications. Total time under general anesthesia was 2 hours and 39 minutes. Around 2 hours postoperatively, while in PACU, the patient developed acute onset respiratory distress with inspiratory stridor. She received a bolus of epinephrine 0.2 mg and her airway was supported with continuous positive airway pressure. Within minutes the symptoms resolved and the patient was admitted to the medical intensive care unit. The patient was also given methylprednisolone 125 mg and started on cetirizine 10 mg every 8 hours and famotidine 20 mg every 8 hours. Postoperative pain was managed with methadone 5 to 10 mg. Approximately 22 hours postoperatively she developed diffuse pruritus followed by barking cough, hoarseness, and chest tightness. A bolus of epinephrine 0.5 mg was given and she was placed on bilevel positive airway pressure with return to respiratory baseline several minutes later. Additional doses of cetirizine, famotidine and methylprednisolone were given. She remained hemodynamically stable and the rest of her postoperative course was uneventful.

## Discussion

### Diagnosis

Current diagnosis of MCAS requires fulfillment of three criteria (***Table 1***)^[2–3]^. First, there must be typical clinical symptoms of mast cell degranulation involving >2 organ systems concurrently in more than one episode. Second, at least two mast cell specific mediators should be elevated. These include plasma tryptase, prostaglandin D2, histamine or urinary N-methylhistamine, leukotriene, 11-β-prostaglandin F2α, and/or increased mast cell numbers in extracutaneous tissue^[2]^. Third, the patient should demonstrate symptomatic response to anti-mediator drugs^[2]^. Failure to respond to targeted treatment may indicate other underlying disease process since other conditions, such as carcinoid syndrome, and pheochromocytoma, may mimic MCAS^[1,4]^.

**Table 1:** Characteristics of mast cell activation syndrome

Symptoms	Triggers	Mediators	Diagnosis	Treatment
Mild							
Flushing Pruritus Bloating Diarrhea	Food products Medications Extreme temperature	Histamine Neutral proteases Proteoglycans	Clinical symptoms of mast cell degranulation involving two or more organ systems concurrently	Minimizing psychological stressors Avoidance of mechanical stressors H1/H2 receptor antagonists Leukotriene receptor antagonists
Moderate/severe							
Hypotension Tachycardia Bronchospasm Angioedema Anaphylaxis	Pain Psychosocial stress Mechanical pressure Hormonal fluctuation	Cytokines (such as: TNF-alpha)	Elevation in at least two mast cell specific mediators Symptomatic response to anti-mediator drugs	Prostaglandin D2 antagonists Cromolyn (not recommended in acute setting) Epinephrine (acute anaphylaxis)

### Symptoms

Symptoms often correlate to the specific mediator being released and reaction severity may depend on the capacity of mast cells to release mediators (***Table 1***)^[4]^. Mild symptoms may include flushing, pruritis, bloating or diarrhea. More severe symptoms may include angioedema, hypotension, tachycardia or anaphylaxis^[4]^. In patients with mastocytosis, anaphylaxis appears more likely to manifest as cardiovascular compromise (*e.g.*, hypotension); angioedema and bronchospasm occur less frequently.

### Triggers

Numerous triggers of mast cell (MC) degranulation have been identified including food products, medications, extreme temperature, pain, psychosocial stress, mechanical pressure, and hormonal fluctuations(***Table 1***)^[1,4]^. The perioperative setting presents a unique challenge for these patients as it exposes them to many of these factors. For example, awaiting surgical intervention can be anxiety provoking and patients often experience different forms of mechanical pressure including intravenous catheter placement and non-invasive blood pressure monitoring with a pneumatic cuff. This holds true for the intraoperative setting, which also exposes the patient to temperature fluctuations and physiological stressors which often translate into hemodynamic alterations leading to autonomic nervous system response and release of different stress hormones (*i.e.*, catecholamines). Throughout the perioperative period, patients are exposed to a number of different medications which retain the potential for MC degranulation.

In the general population, neuromuscular blockers have been shown to cause 60% to 70% of anaphylaxis events intra- and postoperatively, followed by antibiotics^[5]^. Succinylcholine and cisatracurium have demonstrated the lowest MC activation potency, whereas mivacurium and atracurium show the highest potency of MC activation. Penicillin and cephalosporins are the leading causes of antibiotic anaphylaxis, followed by betalactams, vancomycin and quinolones^[6]^. Intravenous anesthetics are generally considered safe, excluding thiopental which has historically been associated with increased risk of anaphylaxis^[6]^. Synthetic opioids such as fentanyl, remifentanil, and buprenorphine demonstrate less MC mediator release than morphine, meperidine and codeine^[7]^. There is no conclusive evidence that regional anesthesia or local anesthetics lead to increased adverse events in patients with MCAS compared to those in the general population^[7]^. One retrospective study of 459 adult patients did demonstrate increased risk of MCAS symptoms in those undergoing more invasive surgeries and receiving GA rather than regional anesthesia or sedation^[8]^.

### Treatment

The goal of treatment of MCAS is to mitigate symptoms and prevent anaphylaxis; this hinges on preventing MC inflammatory mediators from binding their receptors. This can be achieved by preventing MC degranulation and mediator release, or by blocking the receptor itself ^[1]^. For any patient with MCAS, the perioperative goal should be to avoid known triggers, which requires a careful review of previous allergies and reactions. Psychologic stressors should be minimized and a comfortable environment avoiding temperature extremes or loud noises should be maintained. Mechanical factors such as friction and pressure (*i.e.*, tourniquets) should also be avoided or minimized.

### Premedication

It has been reported premedication is helpful to avoid anaphylaxis in these patients^[7–8]^. However, there have been no randomized controlled trials to demonstrate the need for premedication or establish a specific beneficial regimen. Histamine is considered the major mediator of anaphylactic reactions, and prevention of histamine release is preferred over rescue treatment^[1]^. H1 and H2 receptor antagonists (H1A, H2A) demonstrate a synergistic effect with a relatively low side effect profile^[1]^. However, first generation H1 antagonists (*e.g.*, diphenhydramine) have anticholinergic effects which may be undesirable in older patients or those with contraindications to anticholinergics^[9]^. In these patients, second generation H1 antagonists, such as cetirizine, may be preferred.

**Figure 1:**
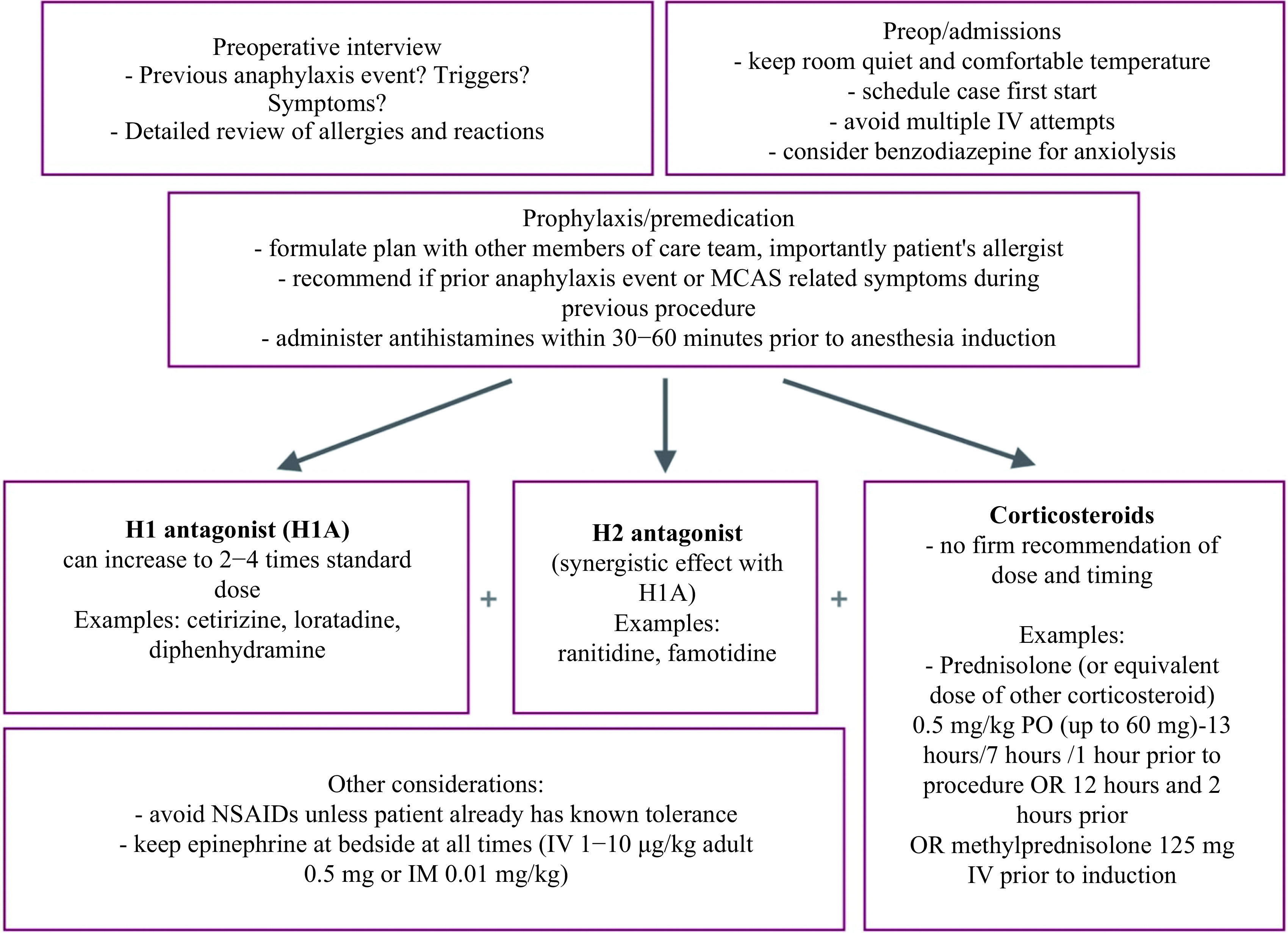
Preoperative pathway for patient with mast cell activation syndrome.

Leukotriene receptor blockers such as montelukast and zafirlukast may be used to treat dermatologic manifestations. Prostaglandin D2 antagonists may help prevent hypotension in some patients; however, most recommend NSAIDs to be only given in those already tolerating them as there have been some reports of increased rates of anaphylaxis in mastocytosis^[1]^. Cromolyn is a MC stabilizing medication with a delayed onset of action and is not typically recommended in the acute setting^[1]^. Although no randomized controlled trial can establish the effectiveness or ideal prophylactic regimen, a proposed preoperative pathway based on the pathophysiology of MCAS and anaphylaxis is outlined below (***Fig. 1***).

### Anaphylaxis

It is estimated that 20% to 50% of patients with systemic mastocytosis will experience systemic anaphylaxis^[1]^. The prevalence in all patients with MCAS is unknown. In the event of acute anaphylaxis, epinephrine should be given either intramuscularly (0.5 mg for patients >60 kg) into the lateral thigh^[10]^ or intravascularly (1–10 μg/kg). This can be repeated every 2 to 5 minutes, and in some cases, patients may require epinephrine infusion. Patients who experience perioperative anaphylaxis should have allergy testing to identify the likely culprit.

In summary, MCAS causes an inappropriate release of inflammatory mediators from MC. These mediators can affect multiple organ systems and lead to significant morbidity/mortality if not recognized and managed appropriately. MCAS triggers can be non-specific and encountered in the perioperative period; therefore, preoperative preparation, trigger avoidance and treatment plans are often crucial for desireable outcomes. If avoidance of specific triggers is not possible, management should include administration of medications targeted at preventing MC degranulation or mediator receptor binding. Specific treatment includes medications such as anti-histamines, leukotriene receptor blockers, steroids, and even epinephrine in the event of anaphylaxis.
